# Swallowing Assessment in Post-Comatose Patients: A Feasibility Study on the SWADOC Tool

**DOI:** 10.3390/jcm13113268

**Published:** 2024-05-31

**Authors:** Roxanne Herr, Amandine Regnier, Marion Belorgeot, Evelyne Mélotte, Jessica Simon, Leandro R. D. Sanz, Nicolas Lejeune, Valérie Chavet, Jenny Paluszkiewicz, Frédéric Pellas, Jean-Baptiste Chevallier, Steven Laureys, Jean-François Kaux, Olivia Gosseries

**Affiliations:** 1 Neurology Department, Haguenau Hospital, 67500 Haguenau, France; orthophonie.herr@gmail.com; 2 Coma Science Group, GIGA-Consciousness, University of Liège, 4000 Liège, Belgium; amandine.regnier@uliege.be (A.R.); evelyne.melotte@uliege.be (E.M.); leandro.sanz@uliege.be (L.R.D.S.); nicolas.lejeune@uliege.be (N.L.); steven.laureys@uliege.be (S.L.); 3 Centre du Cerveau2, University Hospital of Liège, 4000 Liège, Belgium; 4 Physical Medicine and Rehabilitation Medicine Department, University of Liège and University Hospital of Liège, 4000 Liège, Belgium; jfkaux@chuliege.be; 5 Physical Medecine and Rehabilitation Medicine Department, University Hospital of Nîmes, 30029 Nîmes, France; marion.belorgeot@wanadoo.fr (M.B.); frederic.pellas@chu-nimes.fr (F.P.); 6 Psychology and Neuroscience of Cognition Research Unit (PsyNCog), University of Liège, 4000 Liège, Belgium; j.simon@uliege.be; 7 William Lennox Neurological Hospital Center, 1340 Ottignies-Louvain-la-Neuve, Belgium; 8 Institute of NeuroScience, Université Catholique de Louvain, 1200 Brussels, Belgium; 9 Physical Medicine and Rehabilitation Medicine Department, Center for Traumatology and Rehabilitation Erasme, 1070 Brussels, Belgium; valerie.chavet@erasme.ulb.ac.be; 10 Physical Medicine and Rehabilitation Medicine Department, Neurological Center for Functional Rehabilitation, University Hospital of Liège, 4557 Fraiture, Belgium; jpaluszkiewicz@chuliege.be; 11 EVC-EPR Unit, Functional Rehabilitation Center of Fontfroide, 34070 Montpellier, France; jeanbaptiste.chevallier@wanadoo.fr; 12 Joint International Research Unit on Consciousness, CERVO Brain Research Centre, Laval University, Québec, QC G1J2G3, Canada

**Keywords:** swallowing, dysphagia, consciousness disorders, severe brain injury, assessment

## Abstract

**Background:** After a severe brain injury and a coma, patients may develop disorders of consciousness (DoC), frequently accompanied by severe dysphagia. The evaluation and therapy of swallowing are therefore essential aspects of their management. Objectives: This study aims to evaluate the SWallowing Assessment in Disorders of Consciousness (SWADOC) tool in the assessment of swallowing in post-comatose patients. Here, we validate its quantitative items, describe preliminary results and identify limitations. **Methods:** Fourteen post-comatose patients were repeatedly evaluated with the Simplified Evaluation of CONsciousness Disorders (SECONDs) and with the SWADOC. **Results:** The internal consistency of the oral and pharyngeal subscales of the SWADOC was good. The test–retest reliability showed that all items, all subscores and the total score were stable except for two items (endo-buccal secretions and bronchial congestion). A comparison to the Facial Oral Tract Therapy Swallowing Assessment of Saliva (F.O.T.T-SAS) confirmed that scoring with the SWADOC offers a greater potential for quantitative observations in assessing swallowing abilities among patients with DoC. The SECONDs scores and SWADOC total scores showed a significant positive correlation (τ = 0.78, *p* < 0.001). **Conclusions:** This study provides preliminary but encouraging results on the psychometric properties of the SWADOC tool. It shows that this tool is relevant and feasible as a bedside assessment of dysphagia in patients with DoC.

## 1. Introduction

### 1.1. Background 

After a severe brain injury and an initial period in coma, some patients may develop disorders of consciousness (DoC). DoC are a continuum of states ranging from absent awareness and arousal to preserved arousal with fluctuating awareness: coma, unresponsive wakefulness syndrome (UWS), minimally conscious state minus (MCS-) and minimally conscious state plus (MCS+). Patients in UWS can open their eyes but demonstrate only reflex movements [1,2], contrasting with coma patients who keep their eyes closed [3]. Patients in the MCS show inconsistent but reproducible signs of consciousness [4]. More precisely, MCS- defines patients who show non-language-related behavioral responses (e.g., visual pursuit, fixation, localization of noxious stimulation). Patients in MCS+ display language-related behavioral responses, such as command following or intentional communication. When patients recover functional communication or use of objects, they emerge from the MCS (EMCS) [5]. Patients may temporarily occupy one of these states or remain in it indefinitely [6]. The assessment of consciousness is based on validated behavioral scales, which can be complemented with neuroimaging exams. The most comprehensive scale is the Coma Recovery Scale-Revised (CRS-R) [7,8]. The Simplified Evaluation of CONsciousness Disorders (SECONDs) is a shorter, reliable and valid tool [9] developed using the most prevalent signs of consciousness observed with the CRS-R and a communication item.

Dysphagia, also known as swallowing disorders, refers to the impairment of one or more components of the swallowing process, encompassing the mouth, tongue, oral cavity, pharynx, airway and esophagus, along with its upper and lower sphincters [10]. Dysphagia is consistently present in patients with DoC and appears to be associated with level of consciousness [11]. Indeed, some components of swallowing are more often impaired in UWS than in MCS patients: the efficacy of the oral phase, the cough reflex and the presence of a tracheostomy (linked to saliva management). In particular, there is some evidence that patients in UWS never show an efficient oral phase (i.e., adequate lip prehension, tongue propulsion and absence of post-swallowing oral stasis) [11]. These results are of particular interest in the diagnosis of consciousness, as they may lead to the integration of swallowing behaviors into diagnostic criteria. Moreover, the assessment of swallowing disorders in patients with DoC is essential due to their frequent comorbidities (e.g., aspiration pneumonia, malnutrition) and their functional consequences (e.g., tracheostomy to assist ventilation and gastrostomy for nutritional support) [12]. 

**Figure 1:**
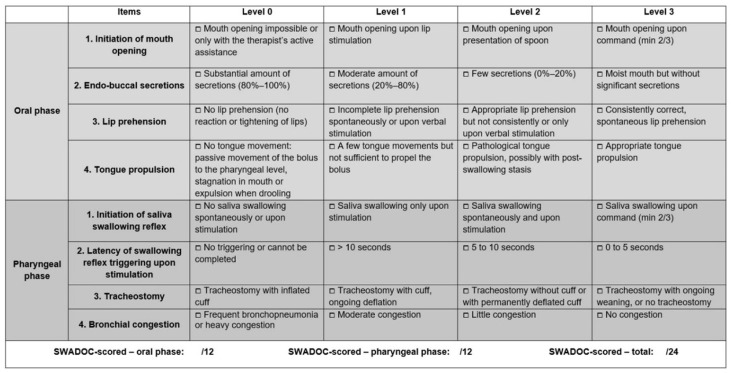
SWADOC-scored assessment. Taken with permission from Mélotte et al. (2021) [18]. While this study employed the French version, we have provided an English translation for clarity. Note that the English version has not yet been validated.

The assessment of swallowing in patients with DoC is challenging, since most of these patients cannot communicate or actively participate. Instrumental methods such as fiber-optic endoscopy or videofluoroscopy are particularly valuable in this population, given their heightened risk of aspiration and fluctuating swallowing abilities, especially in cases where oral feeding, whether partial or complete, is being considered [13,14,15]. However, videofluoroscopy may not always be feasible for all DoC patients, as it requires patient transportation, positioning in an upright posture and the ingestion of a bolus [16], unlike fiber-optic endoscopy, which can be performed at the bedside and allows the observation of spontaneous saliva swallowing [13]. However, therapists involved in dysphagia management need practical behavioral and functional assessments that can be conducted at patients’ bedsides as part of their daily clinical practice. Until recently, there was a serious lack of tools to assess swallowing in patients with DoC, as classical swallowing assessments require patients’ active participation and are unsuitable for non-communicative patients [17,18]. To fill this gap, the SWallowing Assessment in Disorders of Consciousness (SWADOC) was recently developed [18]. It includes 8 quantitative and 50 qualitative items. The quantitative items, each scored from 0 to 3 points, form the “SWADOC-scored” items (Figure 1) and are separated into four oral phase items and four pharyngeal phase items. This enables the calculation of oral and pharyngeal subscores, as well as the total score. The choice of using the SWADOC-scored items or the full SWADOC scale depends on the therapeutic goals. The SWADOC-scored items are sufficient if a therapist wants to quantitatively measure a patient’s progress or the effectiveness of therapy, while the qualitative items allow a more detailed evaluation when the therapist wants to precisely explore the patient’s swallowing abilities.

### 1.2. Study Aims and Hypotheses

The primary objectives of this feasibility study are:

− Assess the suitability of implementing the SWADOC with DoC patients in a clinical environment, as indicated by the potential number of assessments per patient;
− Determine if the SWADOC enables the differentiation of patient swallowing profiles without encountering floor or ceiling effects in the scores;
− Preliminarily validate the SWADOC by assessing its internal consistency and test–retest reliability. Additionally, compare the SWADOC to the F.O.T.T.-SAS, a relevant tool for screening dysphagia;
− Investigate the association between DoC behavioral diagnosis and SWADOC scores;
− Identify potential limitations and propose adjustments to the validation protocol.

## 2. Materials and Methods

### 2.1. Study Design

This study is a multicenter prospective cohort study. The research protocol was reviewed and approved by two central ethics committees (the Ethics Committee of the Faculty of Medicine of the University Hospital of Liège (2020-79) and the Ethics Committee of Île de France XI (20.05.26.70621)), as well as by the ethics committees of the participating hospitals and clinics. The SWADOC was created in French, and we used the French version in this study, which is available in Supplementary Materials File S1.

### 2.2. Population

Patients with DoC (UWS, MCS-, MCS+) or those emerging from DoC (EMCS) following a severe acquired brain injury were enrolled in this study. They were recruited from four inpatient neurological rehabilitation programs in post-coma units and rehabilitation services in Belgium and France (William Lennox Neurological Hospital in Ottignies, the Center for Traumatology and Rehabilitation Erasme in Brussels, the Neurological and Functional Rehabilitation Centre of University Hospital of Liège in Fraiture-en-Condroz and the Functional Rehabilitation Clinic of Fontfroide in Montpellier) between July 2020 and January 2021. For each patient enrolled in this study, informed consent had to be signed by their legal representative. Inclusion criteria were (1) age above 18 years; (2) being a French native speaker or having advanced language skills prior to the injury (i.e., based on the medical history communicated by the family); (3) having experienced a prior coma phase caused by a severe acquired brain injury; (4) medical stability (no mechanical ventilation or sedation, no acute medical pathology such as infection or respiratory distress); (5) no neurological or otorhinolaryngological disease prior to the brain injury that could impact swallowing; (6) a minimum of 28 days since the acquired brain injury at inclusion (i.e., individuals with prolonged DoC); (7) diagnosis of UWS, MCS–, MCS+ or EMCS based on neurobehavioral assessments; and (8) the presence or absence of a tracheostomy.

### 2.3. Intervention and Measures

**Figure 2:**
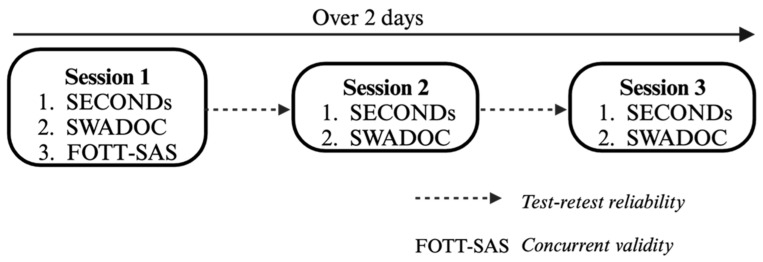
Design of this study.

To validate the SWADOC and explore the potential links between the SWADOC items and level of consciousness, the patients underwent three sessions of assessment of their level of consciousness and of their swallowing abilities. The three sessions took place on two working days (maximum four days apart), with at least one evaluation in the morning and one in the afternoon (Figure 2). The structure of the protocol was based on previous studies, specifically the validation studies of the SECONDs [9] and the BERA [19]. All assessments were conducted by a single investigator (R.H.), who had been trained to administer the different scales. Consciousness and swallowing were assessed with three different tools:

1. SECONDs [9]: this tool is a brief (the administration time is approximately 7 min), reliable and valid 8-item scale, ranging from 0 to 8, that is directly related to consciousness diagnosis (0 = coma, 1 = UWS, 2–5 = MCS-, 6–7 = MCS+, 8 = EMCS) [9]. We chose to use this rapid scale to prevent excessive fatigue for the patients. Through the utilization of the SECONDs, a validated behavioral measure of consciousness level, our objective was to explore the potential association between swallowing and consciousness. By administering both scales concurrently, we aimed to investigate the relationship between swallowing function and level of consciousness.
2. SWADOC [18]: this tool includes 56 items, among which 8 quantitative items score from 0 to 3 points each, that constitute the “SWADOC-scored” items (Figure 1). The duration of the SWADOC assessment varies from 10 to 30 min depending on patients’ degrees of arousal and on their abilities. Oral and pharyngeal subscores (ranging from 0 to 12) and a total score (ranging from 0 to 24) can be calculated. The SWADOC mainly investigates salivary swallowing but also includes a functional test with 5 mL of thickened water, which is performed only if a spontaneous or stimulated swallowing reflex event is observed before and if the patient has no bite reflex or lockjaw. The complete version of the SWADOC’s administration guide is available in the protocol study [18].
3. Facial Oral Tract Therapy Swallowing Assessment of Saliva (F.O.T.T.-SAS) [20,21]: this test is based on 7 items. The assessment points include: 1. Conscious and/or response to verbal address; 2. Able to sit upright with some help control; 3. Oral transport of saliva; 4. Spontaneous or facilitated swallowing of saliva, 5. Coughing following swallowing of saliva; 6. Gurgling breath sound following swallowing of saliva; 7. Difficulties breathing following swallowing of saliva. If the first four items are answered by “Yes” and the next three are answered by “No”, oral intake can be initiated.

The SECONDs was administered before the SWADOC, and the F.O.T.T.-SAS items were completed during the first SWADOC assessment. If the patient fell asleep during the assessments, the examiner could perform at maximum three arousal protocols (tactile stimulations) similar to the CRS-R [7]. Repeating three assessment sessions over 2 working days allowed the assessment of the test–retest reliability of each item in the SWADOC. It also reduced the risk of consciousness misdiagnosis by repeating the SECONDs assessments, as the SECONDs was previously validated with three assessments [9]. The diagnosis of consciousness was determined by the best SECONDs score within the three assessments for each patient.

Information about age, gender, time since injury, etiology and type of feeding were also collected from the patients’ medical records. The type of daily feeding was measured with the Food Intake Level Scale (FILS), an observer-rated 10-level scale of type of feeding [22].

### 2.4. Data Analysis

Demographic data were described by number (%), mean (and standard deviation (SD)) and/or median (and interquartile range (IQR)). The internal consistency of the items was evaluated by Cronbach’s alpha, calculated for each subscale (oral phase and pharyngeal phase) and each assessment session. A 0.70 score was required as the minimal alpha for acceptable internal consistency. The test–retest reliability of each item, subscore and total score was calculated with Kendall’s tau-b between the first and the second assessment sessions, between the first and the third assessment sessions and between the second and the third assessment sessions, using the measures from the patients who completed all the assessments. A value greater than 0.50 ensured the stability of ratings over the three evaluations. To describe the association between consciousness levels and swallowing abilities, we used Kendall’s correlation between the best SWADOC total score and the associated SECONDs score from the same session. A descriptive analysis was conducted to examine the relationship between SWADOC scores and level of consciousness, as well as to compare the SWADOC to the F.O.T.T.-SAS. This analysis involved calculating the means and medians of item scores and subscores. Statistical analyses were made using Jamovi v1.6.15 and R v 4.2.2. 

## 3. Results

### 3.1. Demographic and Clinical Characteristics

Fourteen patients were included in this study and consisted of three UWS, five MCS-, four MCS+ and two EMCS patients (six traumatic brain injuries; three anoxic and five non-traumatic brain injuries; five women; median age = 56.5 years, interquartile range (IQR) = 13.75 years; median time since injury = 12.3 months (IQR: 90.72 months)). Among the 14 patients, three UWS, two MCS- and two MCS+ patients had tracheostomies by the time of the assessments. The demographic and clinical characteristics along with the detailed SECONDs and SWADOC scores, are provided in Table 1 by group and in the Supplementary Materials individually (Supplementary Materials Table S1). For three patients, the third assessment session could not take place because of their low levels of arousal (one patient did not open their eyes despite three arousal protocols) or medical complications not compatible with the assessment (one had emesis and one had abdominal pain, according to the medical team). The SECONDs score was stable for 12 among the 14 patients (three UWS, four MCS-, three MCS+ and two EMCS). Two patients showed a change in diagnosis over the three SECONDs assessments (MCS- to MCS+, UWS to MCS-). Finally, two other patients had variations in their SECONDs scores (6—response to command or 7—intentional communication) without a change in diagnosis (MCS+), showing fluctuations in their ability to communicate.

The time required for the SWADOC assessment ranged between 10 and 30 min depending on the patients’ degrees of arousal and on their abilities (e.g., functional tests were not performed on patients who were unable to swallow spontaneously or upon stimulation or if they presented a bite reflex or lockjaw).

### 3.2. Preliminary Measures of the Validity of the SWADOC-Scored Items

#### 3.2.1. Internal Consistency

Across the three assessment sessions (Table 2), the internal consistency of the oral subscale resulted in an α value between 0.78 and 0.80, indicating good internal consistency. For the pharyngeal subscale, the α value ranged between 0.58 and 0.77, indicating that its internal consistency was close to the acceptable value depending on the assessment sessions. When the P3 item (tracheostomy) was removed, the α value of the pharyngeal subscale increased from 0.73 to 0.79. Similarly, removing the P4 item (bronchial congestion) also led to an increase in the α value, from 0.70 to 0.79. These results can be explained by the fact that the P3 and P4 items refer to the efficacy of the pharyngeal phase, while the P1 (initiation of saliva swallowing reflex) and P2 (latency of swallowing reflex triggering upon stimulation) items refer to the ability to trigger the pharyngeal phase. The α values for both the oral and the pharyngeal subscales increased across the assessment sessions.

#### 3.2.2. Test–Retest Reliability

Kendall’s tau-b was calculated for each item based on the eleven patients who underwent the three assessment sessions (Table 2). The O1 (initiation of mouth opening) and P3 (tracheostomy) items never changed within the three SWADOC-scored assessments of a patient. The O3 (lip prehension), O4 (tongue propulsion) and P1 (initiation of saliva swallowing reflex) items, as well as the two subscores and the total score, had tau-b values above 0.5. The P2 (latency of swallowing reflex triggering upon stimulation) item was also above 0.5 but closer to the 0.5 value (Kendall’s tau-b = 0.53; 0.77; 0.67). The O2 (endo-buccal secretions) and P4 (bronchial congestion) items were subject to test–retest variations (O2 Kendall’s tau-b = 0.47; 0.40; 0.87 and P4 Kendall’s tau-b = 0.65; 0.65; 0.39). Only one patient (EMCS) among the 14 had exactly the same score for every item in the three SWADOC-scored assessments.

#### 3.2.3. Comparison between the SWADOC and the F.O.T.T.-SAS

Among the 14 patients, 13 had a level of “No, oral intake should not be initiated” on the F.O.T.T-SAS, with SWADOC scores ranging from 6 to 21 (mean of 13.4) and FILS scores from 1 to 2. The only patient with a level of “Yes, oral intake should be initiated” was EMCS, was already able to feed himself entirely orally (FILS level 9) and had a SWADOC score of 20 (out of 24).

### 3.3. Association between Levels of Consciousness and SWADOC

**Figure 3:**
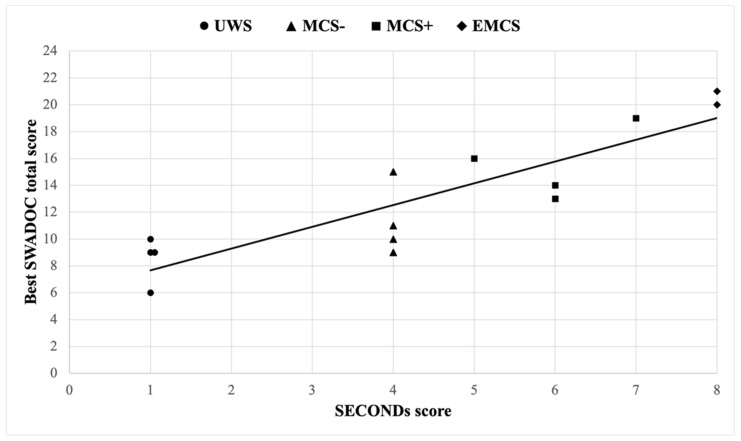
Correlation between each best SWADOC total score and the associated SECONDs score from the same session.

The analysis of the correlation between the best SWADOC total score and the associated SECONDs score from the same session resulted in a Kendall’s tau-b value of 0.78 (*p* < 0.001), indicating that SECONDs and SWADOC scores are strongly positively correlated (Figure 3). 

**Figure 4:**
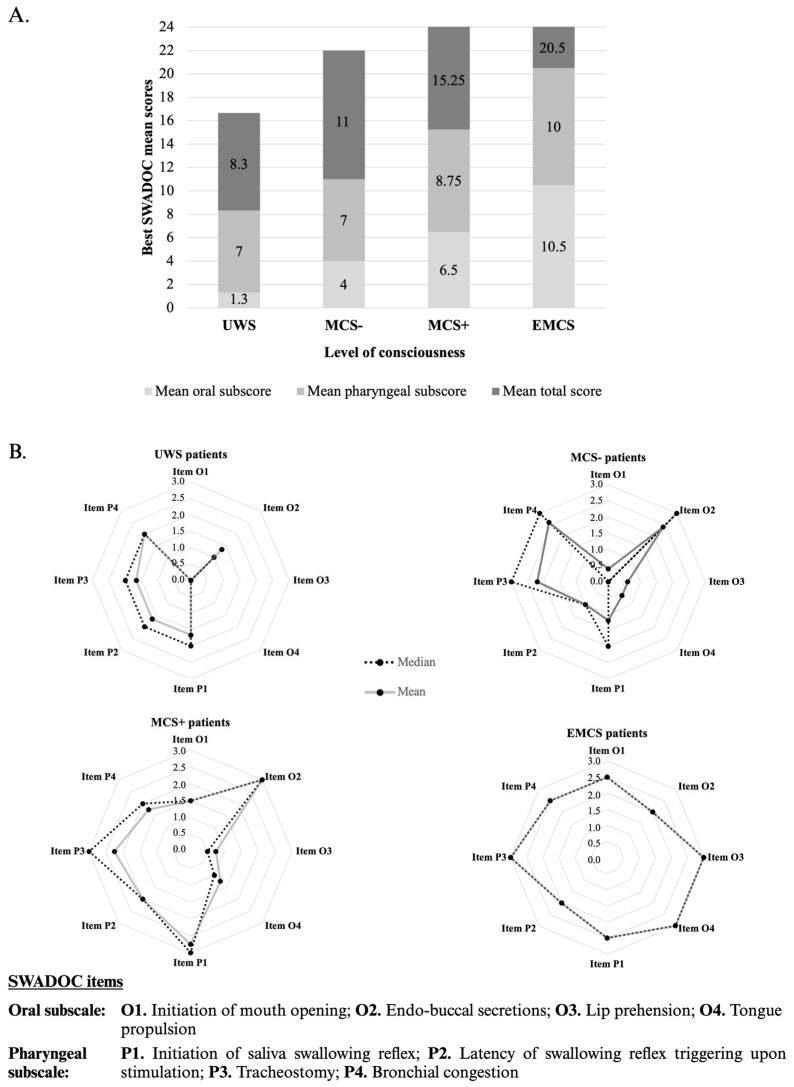
Mean oral and pharyngeal subscores and mean total score (**A**) and mean and median item scores (**B**) for UWS, MCS-, MCS+ and EMCS patient groups.

A descriptive analysis of the association between level of consciousness and the SWADOC-scored items and subscores is described in Figure 4. The levels of consciousness were positively associated with the oral subscore and the oral items O1 (initiation of mouth opening), O3 (lip prehension) and O4 (tongue propulsion), corresponding to the effectiveness of the oral phase. Indeed, the three UWS patients (100%) obtained a mean oral subscore of 1.3 (mean score of 0 at O1, O3, O4). The MCS- patients had a mean oral subscore of 4 (mean scores of 0.4 at O1, 0.6 at O3, 0.6 at O4), while the MCS+ patients had a mean oral subscore of 6.5 (mean scores of 1.5 at O1, 0.75 at O3, 1.25 at O4). The EMCS patients had a mean oral subscore of 10.5 (mean scores of 2.5 at O1, 3 at O3, 3 at O4). On the other hand, the association between the pharyngeal subscores and the level of consciousness seemed to be less pronounced, with mean pharyngeal subscores of 7 for UWS, 7 for MCS-, 8.75 for MCS+ and 10 for EMCS patients.

Concerning the qualitative items, no influence of level of consciousness was noticed except for the item of the swallowing reflex triggering with a small amount of thickened water (5 mL). Indeed, the functional test was proposed just once, to one out of the three UWS patients, but no swallowing reflex was triggered, and it was not feasible in two out of the five MCS- patients. Among the three MCS- patients who underwent the functional test, one did not trigger a swallowing reflex at all, while the two others did. Finally, all MCS+ and EMCS patients could perform the functional test, and all of them did trigger the swallowing reflex.

## 4. Discussion

In this study, we evaluated the clinical application of a new tool, the SWADOC, which allows the assessment of swallowing abilities in patients with DoC. The preliminary data demonstrated good internal consistency and test–retest reliability, showing that this tool is feasible and can be adapted to the daily assessment of patients with DoC.

### 4.1. SWADOC’s Relevance and Benefits

#### 4.1.1. Relevance and Feasibility in a Clinical Setting

To date, there is no specific validated tool to assess swallowing function in post-comatose patients [11]. Among the existing tools, the F.O.T.T-SAS stands out as particularly relevant to our population of interest, as it also addresses consciousness and arousal [23]. This tool enables a rapid screening of dysphagia through an assessment of seven items, aimed at determining whether oral intake is feasible for patients with severe acquired brain injury. The F.O.T.T.-SAS has a dichotomous outcome, intended to distinguish patients in two categories: “Oral intake should be initiated” or “Not initiated” [21]. However, this dichotomous outcome is not suitable for our population, as most patients with DoC typically depend on artificial nutrition and frequently face challenges in transitioning from artificial nutrition to partial oral feeding and, ultimately, to full oral feeding [11]. Hence, there is a need to create a specialized tool to document dysphagia at the bedside in patients with DoC, and we propose that the SWADOC could suitably perform this role.

In this feasibility study, the comparison between the F.O.T.T-SAS and SWADOC revealed that among the 14 patients included in this study, 13 received a “No oral intake indication” from the F.O.T.T-SAS, while the SWADOC total scores ranged from 4 to 21. This suggests that the F.O.T.T-SAS may not adequately differentiate between swallowing profiles, unlike the SWADOC, which offers insight into the efficacy of both the oral and pharyngeal phases of swallowing. However, it is essential to note that while a high score on the SWADOC is indicative, it does not guarantee the safe reintroduction of oral feeding. The SWADOC was developed with the aim of assisting therapists in better defining swallowing disorders and the therapeutic needs of patients with DoC. Therefore, SWADOC assessments alone are insufficient in indicating the reintroduction of oral feeding but are complementary to other swallowing assessments using instruments (fiber-optic endoscopy or videofluoroscopy if indicated) for patients at high risk of aspiration [11,13].

In addition to this, the feasibility of the SWADOC in a clinical setting is also a point of interest. Indeed, this tool is a non-invasive bedside assessment, requiring only a short amount of time (10 to 30 min). Moreover, 93% of the assessments were completed as scheduled (39 among 42 evaluations), which suggests that the SWADOC is also suitable for post-comatose patients despite their high arousal fluctuations.

#### 4.1.2. Internal Consistency

While the internal consistency of the oral subscale was good, the pharyngeal subscale fluctuated over the acceptability threshold depending on the assessment sessions. The lack of consistency of the pharyngeal items can be explained by the heterogeneity of the items. Indeed, P1 (initiation of the swallowing reflex) and P2 (latency of the swallowing reflex upon stimulation) refer to the ability to trigger a swallowing reflex, while P3 (tracheostomy) and P4 (bronchial congestion) refer to the efficacy of the pharyngeal phase. It will be interesting to analyze the internal consistency in a larger population. If the results remain consistent, it may be worth considering separating the pharyngeal phase into two subscores: “ability to trigger the swallowing reflex” and “efficacy of the pharyngeal phase”.

#### 4.1.3. Test–Retest Reliability

The preliminary analysis of the test–retest reliability shows that while some items are very stable (O1, O3, O4, P1, P3), others vary substantially over time (O2, P4 and P2 in a lesser measure). During the assessments, we noticed that some quantitative items seemed to depend partly on external factors. For instance, the amounts of endo-buccal secretions (O2 item) or of bronchial congestion (P4 item) were partly dependent on the time since the last tracheostomy suctioning or mouth care by a health care team or the last respiratory physiotherapy session. Moreover, those interventions are not performed the same way across hospitals and clinics. We suggest a higher standardization of the external conditions described, e.g., by providing, if required, mouth care and/or tracheostomy suctioning and respiratory physiotherapy one hour before the SWADOC assessment, such that during the evaluation, patients will have amounts of endo-buccal secretions and degrees of congestion representative of their swallowing abilities. Nevertheless, the important arousal fluctuations of patients with DoC are well-described and probably require a repetition of SWADOC assessments in order to gain insight on the fluctuations of their swallowing abilities and base therapy on the most critical situations for the patient’s safety. 

Fluctuations in patients’ levels of arousal can introduce variability in their performance on swallowing assessments, thereby affecting both internal consistency and test–retest reliability. These fluctuations may result in inconsistent responses and scores across different assessment sessions. Consequently, determining the precise number of assessments required should be a subsequent step in the validation procedure.

#### 4.1.4. Links between Consciousness and Swallowing

The results concerning the association of levels of consciousness with the SWADOC total scores are in line with previous findings [10] showing that the voluntary part of swallowing, i.e., the oral phase, is associated with the level of consciousness. Moreover, as the three UWS patients (100%) had 0 points on items O1, O3 and O4 (initiation of mouth opening, lip prehension and tongue propulsion), this supports the identification of some swallowing components (especially oral ones, such as the efficiency of the oral phase) as signs of consciousness that could be integrated in diagnostic criteria, as other studies suggest [11]. The presence and efficiency of certain components, as well as their evolution over time, measured with the SWADOC, could also potentially be prognostic markers of a patient’s recovery [24]. 

The behavioral scales utilized for patient diagnosis encompass auditory, visual and other sensory pathways but do not incorporate elements related to swallowing. Nevertheless, in few cases, some patients may be categorized as UWS due to the absence of behavioral responses through this behavioral assessment despite maintaining intact swallowing abilities [25]. Therefore, integrating swallowing-related components could enhance consciousness diagnosis, reduce diagnostic uncertainty or at least corroborate findings.

### 4.2. Limitations of This Study

Due to the specificity of this population, the primary limitation of this feasibility study is the small sample size of participants. Additionally, there is a considerable variability in time since injury across patients and the heterogeneous nature of brain injury etiologies could potentially impact patient prognosis. The results presented here should be taken as preliminary data that need to be confirmed in larger samples. In addition, for three patients, the third assessment was not performed because of important arousal fluctuations. To address this issue in the upcoming large-scale validation study, we recommend extending the timeframe for conducting the baseline evaluation with the CRS-R and the three assessment sessions to more than 2 days. 

For this feasibility study, we were limited to only one investigator to conduct the assessments with the patients due to resource constraints. This prevented estimation of the interrater reliability. 

Regarding the order of administration of the different assessments, the evaluation of consciousness level was always performed before the swallowing assessment because it was faster to administrate. As a result, we did not anticipate a notable effect on fatigue, although it is a potential consideration within our population. 

Finally, the results obtained are inherently tied to the French-speaking population. However, despite this linguistic specificity, the implications of our study extend beyond this population. There is a significant need for standardized and validated assessment tools for evaluating swallowing difficulties in DoC patients across various linguistic contexts.

Following the completion of this feasibility study, we have refined our protocol in response to the limitations identified. The validation protocol for the SWADOC has been published, incorporating several enhancements [18]. Firstly, an extension of the testing period was implemented to facilitate the completion of all planned assessments for patients and to enhance flexibility within the clinical context. Assessments now occur over a five-day period for the baseline evaluation with the CRS-R and the three subsequent assessment sessions. Additionally, randomization of the order between consciousness and swallowing assessments was adopted, as it represents a more robust design and addresses potential fatigue effects on patients. To validate inter-rater reliability, a second investigator administers the SWADOC during the same morning or afternoon, either preceding or following the first investigator.

## 5. Conclusions

This feasibility study supports the benefits of the SWADOC, which was developed to address a lack of comprehensive and adapted tools that assess swallowing in patients with DoC. It shows that the SWADOC is applicable at the patient’s bedside, is time-efficient and could be administered in 93% of these cases. The preliminary results show that the SWADOC-scored results were stable across evaluations, except for the O2 and P4 items (i.e., endo-buccal secretions and bronchial congestion). Moreover, the SWADOC-scored items had good internal consistency. The comparison to the F.O.T.T-SAS indicated that the SWADOC offers a greater potential for quantitative observations in assessing swallowing abilities in patients with DoC. A strong positive correlation between the SWADOC total score and the SECONDs score, as well as an association between SWADOC scores and the levels of consciousness, was also described. These results seem to support the presence of a link between the oral phase components of swallowing and consciousness and suggest that some swallowing components may be considered signs of consciousness. Further studies are needed to validate the SWADOC on a larger scale, thus allowing a more accurate bedside clinical assessment of swallowing and a better understanding of the relationships between consciousness and swallowing components.

## Figures and Tables

**Table 1:** Demographic characteristics of the patient group enrolled in this study.

Diagnosis	Patients, n (%)	Gender, Male/ Female	Age, Years, Median (IQR)	TSI, Months, Median (IQR)	Etiology: Traumatic (T), Anoxic (A), Vascular (V), Epileptic (E)	FILS Score, Median (IQR)	SECONDs’ Best Score, Median (IQR)	Oral Subscore, Median (IQR)	Best SWADOC Pharyngeal Subscore, Median (IQR)	Total Score, Median (IQR)
UWS	3 (21%)	1/2	55(12)	87.86 (131.64)	T: 2 V: 1	1 (0)	1 (0)	1 (1)	1 (1)	9 (4)
MCS-	5 (36%)	3/2	65 (12)	103.37 (81.5)	T: 3 V: 3	1 (0.5)	4 (1)	4 (4)	7 (4)	10 (4.5)
MCS+	4 (29%)	3/1	60 (12.5)	8.63 (6.11)	T: 2 V: 1 A: 1	2 (0.75)	6.5 (1)	6 (5.5)	9.5 (4.75)	14.5 (4.75)
EMCS	2 (14%)	2/0	Mean: 45.5 (SD: 5)	Mean: 12.2 (SD: 2)	T: 1 E: 1	Mean: 6 (SD: 4.24)	Mean: 8 (SD: 0)	Mean: 10.5 (SD: 0.7)	Mean: 10 (SD: 1.4)	Mean: 20.5 (SD: 0.7)
Total	14 (100%)	9/5	56.5 (13.75)	12.3 (90.72)	T: 6 V: 3 A: 4 E: 1	1.5 (1)	5 (3.75)	4 (6.75)	8.5 (3.5)	12 (7.75)

**Table 2:** SWADOC’s internal consistency measured with Cronbach’s alpha and test–retest reliability measured using Kendall’s correlation.

		Cronbach’s α		
Internal consistency	Subscale statistics	AS1	AS2	AS3
	Oral phase subscale	0.78	0.80	0.80
	Pharyngeal phase subscale	0.58	0.72	0.77
		Kendall’s tau-b (*p*-value)		
	Items	AS1–AS2	AS1–AS3	AS2–AS3
Test–retest reliability	O1—Initiation of mouth opening	1 (<0.001)	1 (<0.001)	1 (<0.001)
	O2—Endo-buccal secretions	0.47 (0.102)	0.40 (0.159)	0.87 (0.003)
	O3—Lip prehension	1 (<0.001)	0.83 (0.007)	0.83 (0.007)
	O4—Tongue propulsion	0.82 (0.003)	0.81(0.003)	0.74 (0.009)
	Oral subscore	0.72 (0.004)	0.67 (0.007)	0.90 (<0.001)
	P1—Initiation of saliva swallowing reflex	0.80 (0.003)	0.90 (<0.001)	0.81 (0.003)
	P2—Latency of swallowing reflex triggering upon stimulation	0.53 (0.056)	0.77 (0.008)	0.67 (0.018)
	P3—Tracheostomy	1 (<0.001)	1 (<0.001)	1 (<0.001)
	P4—Bronchial congestion	0.65 (0.024)	0.65 (0.025)	0.39 (0.167)
	Pharyngeal subscore	0.68 (0.006)	0.86 (<0.001)	0.59 (0.017)
	Total score	0.77 (0.001)	0.94 (<0.001)	0.80 (<0.001)

## Data Availability

Data can be shared upon reasonable request to the corresponding author.

## Supplementary Materials

The following supporting information can be downloaded at: https://www.mdpi.com/article/10.3390/jcm13113268/s1, Table S1: Individual demographic and clinical characteristics including SECONDs and detailed SWADOC scores; File S1: French version of the SWADOC's administration guide [7,9,22,26,27,28,29,30].
