# Supplementary material for: Swallowing Assessment in Post-Comatose Patients: A Feasibility Study on the SWADOC Tool
Source: J Clin Med. 2024 May 31;13(11):3268. doi: 10.3390/jcm13113268 (PMC11173236; doi:10.3390/jcm13113268)
Supplement: Supplementary file 1 [file jcm-13-03268-s001.zip › jcm-2964910-FileS1.pdf]

File S1 - French version of the SWADOC's administration guide.

Guide de passation

L'outil SWADOC est un protocole d'évaluation de la déglutition adapté aux patients en état de conscience altérée (ECA). Il a été conçu suite au constat du manque d'outils adaptés aux spécificités des patients ECA (absence de réponse à la commande pour certains et absence de communication fonctionnelle) permettant d'apprécier une série de composants en lien avec la déglutition. L'outil SWADOC a pour objectif d'explorer certains composants en lien avec la phase orale et pharyngée de la déglutition ainsi qu'une série de prérequis et autres composants en lien avec la déglutition. Il ne permet pas de statuer à lui seul sur la possibilité d'une reprise alimentaire, partielle ou totale. Une telle décision auprès des patients ECA ne peut, à notre avis, être prise qu'après un examen objectif de la déglutition (vidéofluoroscopie ou nasofibroscopie de la déglutition).

L'évaluation est non-invasive et utilisable en pratique clinique au chevet des patients, en évaluation initiale puis de manière répétée pour suivre l'évolution du patient.

Ce protocole pourra être utilisé par les cliniciens intervenant auprès des patients ECA, comme une base d'analyse à la fois quantitative et qualitative de leur déglutition, utile dans la formulation de leur projet thérapeutique. De plus, les items quantitatifs (SWADOC-scored) permettent de calculer un score global et des sous-scores de la phase orale et de la phase pharyngée, qui pourront constituer une ligne de base à l'évaluation de l'efficacité d'une prise en charge auprès du patient.

Ce guide comprend : 1) Une série d'informations générales préalables à l'évaluation 2) Le matériel nécessaire, 3) Les éléments à relever lors de l'anamnèse, 4) Quelques spécificités concernant la cotation des items quantitatifs, 5) La grille SWADOC-scored (items quantitatifs uniquement). Vous trouverez ensuite le tableau reprenant les consignes de passation et de cotation de chaque item, qualitatif comme quantitatif. Enfin, vous trouverez en annexe des précisions sur les tests ou protocoles cités, ainsi que les références bibliographiques.

1. Remarques générales

- Il est préférable que le patient soit en position assise lors de l'évaluation
- Au fil de la passation de l'évaluation, le thérapeute informera le patient sur chaque stimulation qui lui est proposée
- Avant de commencer l'évaluation du patient, il convient de s'assurer qu'aucun des éléments suivants n'est présent. Si un de ces éléments est présent, il convient de reporter l'évaluation. De même, si un de ces éléments apparaît au cours de l'évaluation, il convient d'arrêter l'évaluation : fièvre, infection en cours, désaturation en oxygène, instabilité de la fréquence cardiaque, crise neurovégétative, impossibilité à stimuler le patient pour qu'il reste éveillé (on réalisera au maximum 3 protocoles d'éveil lors d'une évaluation)

2. Matériel nécessaire à l'évaluation

- 2 chronomètres 🕓
- Lampe de poche
- Cuillère à café
- Coton-tige imbibé d'une solution sucrée et froide
- 5 mL de solution colorée épaissie froide texture IDDSI3 (modérément épais)[1]

3. Items quantitatifs

- Le niveau 3 est la norme attendue. Plus le score global est élevé, plus les capacités de déglutition du patient sont préservées.
- Un patient à un instant « t » ne peut pas se trouver dans plusieurs niveaux différents pour un même item. Dans le cas où un patient valide plusieurs niveaux, on lui accorde le niveau le plus élevé lors de la cotation (par exemple, si un patient est capable d'ouvrir la bouche à l'approche de la cuillère –niveau 2- et sur commande –niveau 3-, on cote le niveau 3)
- Pour calculer le score global et les sous-scores : compter 3 pour chaque sous-item de niveau 3, 2 pour chaque item de niveau 2, 1 pour chaque item de niveau 1 et 0 pour chaque item de niveau 0. Additionner le score de chaque item de la phase orale pour calculer le sous-score oral, additionner le score de chaque item de la phase pharyngée pour calculer le sous-score pharyngé, et additionner les sous-scores oral et pharyngé pour calculer le score global.

4. Anamnèse

Les informations requises pour l'anamnèse sont à recueillir dans le dossier du patient, ou auprès de l'équipe soignante.

**Date et heure de passation** :<br>**Nom et prénom du patient :**<br>**Sexe** :<br>**Date de naissance** :<br>**Endroit où réside le patient actuellement** :<br>**Suivi logopédique** : oui / non -<br>**Fréquence** :<br>- **Lieu** : en centre / à domicile -<br>**Type** :	**Statut nutritionnel** :<br>- Poids actuel :<br>- IMC (poids/taille²) :<br>- Perte ou prise de poids au cours du dernier mois : .... - au cours des 6 derniers mois : …....<br><br>**Score à la FILS**[2] *(voir annexe n°1)* et niveau correspondant : …………<br>Pour un score FILS inférieur à 7, préciser le **mode d'alimentation entérale** : ☐ gastrostomie ☐ jéjunostomie ☐ sonde naso-gastrique<br>Pour un score FILS supérieur à 3, préciser le **score IDDSI**[1] (ou équivalent -*voir annexe n°2*) **pour les aliments** : ……….

**Date de l'accident** : **Type d'accident** :<br>☐ Vasculaire<br>☐ Traumatique<br>☐ Anoxique<br>☐ Autre :<br><br>**Localisation de l'atteinte cérébrale** :<br><br>**Score de Glasgow au moment de l'accident** :<br>- Ouverture des yeux :<br>- Réponse verbale :<br>- Réponse motrice :	**Aide requise lors des repas** : oui / non – si oui par qui : …………………………<br>**Hydratation** : ☐ entérale ☐ per os<br>En cas d'hydratation per os, préciser le **score IDDSI**[1] **pour les liquides** :<br><br>**Antécédents ORL préalables à l'accident** : oui / non – préciser :<br>…………………………………………………………………<br>**Affections ORL depuis l'accident** : oui / non – préciser :<br>…………………………………………………………………<br>**Infections pulmonaires en cours ou récentes** : oui / non – préciser :<br>…………………………………………………………………<br><br>**Prise en charge en kinésithérapie respiratoire** : oui / non – si oui, préciser la fréquence des séances :<br>…………………………………………………………………<br><br>**Médication pouvant affecter :** ☐ l'état de conscience ☐ la déglutition ☐ la salivation – préciser :<br>…………………………………………………………………<br><br>**Hypersalivation** : oui / non – **Traitement de l'hypersalivation** : ☐ toxines ☐ patchs<br><br>Patient capable de produire : ☐ sons spontanés☐ mots☐ communication fiable **Code de communication** : oui / non – préciser :<br>…………………………………………………………………<br><br>**Scores à la SECONDs**[3,4] **ou à la CRS-R**[5] au moment de l'examen, et niveau correspondant :<br>……………………………………….

5. Grille de présentation des items quantitatifs (SWADOC-scored)

	Items	Niveau 0	Niveau 1	Niveau 2	Niveau 3
**Phase orale**	**1. Initiation d'ouverture buccale**	☐ Ouverture de la bouche impossible ou seulement sur aide active du thérapeute	☐ Ouverture buccale sur stimulation labiale	☐ Ouverture buccale à l'approche de la cuillère	☐ Ouverture buccale sur commande (min 2/3)
	**2. Sécrétions endo-buccales**	☐ Sécrétions en quantité significative (80-100%)	☐ Sécrétions en quantité modérée (20-80%)	☐ Peu de sécrétions (020%)	☐ Bouche humide mais sans sécrétions significatives
	**3. Préhension labiale**	☐ Aucune préhension labiale (pas de réaction ou serrage des lèvres)	☐ Préhension labiale incomplète en spontané ou sur stimulation verbale	☐ Préhension labiale adéquate mais nonsystématique ou uniquement sur stimulation verbale	☐ Préhension labiale correcte et spontanée systématiquement
	**4. Propulsion linguale**	☐ Aucun mouvement lingual : passage passif du bolus au niveau pharyngé, stagne en bouche ou ressort en bavage	☐ Quelques mouvements linguaux mais insuffisants pour propulser le bolus	☐ Propulsion linguale pathologique avec présence possible de stases postdéglutition	☐ Propulsion linguale adéquate
**Phase pharyngée**	**1. Initiation du réflexe de déglutition salivaire**	☐ Pas de déglutition de salive que ce soit sur stimulation ou spontanément	☐ Déglutition de salive uniquement sur stimulation	☐ Déglutition de salive spontanée et sur stimulation	☐ Déglutition de salive sur commande (min 2/3)
	**2. Latence de déclenchement du réflexe de déglutition sur stimulation**	☐ Pas de déclenchement ou nonréalisable	☐ > 10 sec	☐ Entre 5 et 10 secondes	☐ Entre 0 et 5 secondes
	**3. Trachéotomie**	☐ Trachéotomie avec ballonnet gonflé	☐ Trachéotomie avec ballonnet en cours de sevrage	☐ Trachéotomie sans ballonnet ou avec ballonnet dégonflé en permanence	☐ Trachéotomie en cours de sevrage, ou absente
	**4. Respiration et encombrement haut et bas**	☐ Bronchopneumonies fréquentes ou encombrement important	☐ Encombrement modéré	☐ Peu d'encombrement	☐ Pas d'encombrement
**SWADOC-scored phase orale: /12**		**SWADOC-scored phase pharyngée: /12**		**SWADOC-scored total: /24**	

6. **<u>Protocole d'évaluation de la déglutition chez les patients en état de conscience altérée</u>**

Enchaînement de l'examen	Consignes	Notation (items qualitatifs – items quantitatifs phase orale – items quantitatifs phase pharyngée)	Matériel
**1. Eveil**	En entrant dans la chambre, vérifier l'éveil du patient **:**<br>→ Si le patient est éveillé, se présenter et commencer l'évaluation : **Saluer le patient** oralement en utilisant son prénom et en touchant sa main ou son bras. **Se présenter** et expliquer la raison de notre présence : par exemple, « Bonjour, je m'appelle… Je suis logopède/orthophoniste. Je viens voir comment vous allez et vous proposer quelques stimulations et exercices. »<br>→ Si le patient sommeille, essayer de le réveiller en utilisant n'importe quel type de stimulation sensorielle : changer sa position, la luminosité de la pièce, le toucher, lui parler, etc. Si cela ne fonctionne pas, utiliser le protocole d'éveil de la CRS-R[5] *(voir annexe n°3).*<br><br>A la fin de l'évaluation, noter dans quelle mesure le patient a gardé les yeux ouverts.	**Temps d'éveil** : ◻ 0-25% ◻ 25-50% ◻ 50-75% ◻ 75-100%<br><br>**Stimulations nécessaires** : ◻ Oui ◻ Non<br>Si oui :<br>◻ Auditives – préciser :<br>………………………………………………………………………<br>◻ Tactiles – préciser :<br>………………………………………………………………………<br>◻ Visuelles – préciser :<br>………………………………………………………………………	
**2. Position au repos de la tête, des yeux, des masséters et des lèvres**	**Position au repos** : noter la position de la tête, des lèvres et des masséters « au repos » ainsi que la présence éventuelle d'une paralysie faciale ou labiale, et l'ouverture des yeux<br><br>Si des membres de l'équipe soignante ou la famille sont présents, demander si c'est la position de la tête « habituelle ».<br><br>Se positionner du côté où le regard est orienté d'après la position de la tête.	→ **Position du patient** : ◻ au lit ◻ au fauteuil<br>→ **Position de la tête « au repos » :**<br>- *Verticalité* : ◻ Neutre ◻ Flexion ◻ Extension<br>- *Horizontalité* : ◻ Neutre ◻ Rotation D ◻ Rotation G<br>◻ Inclinaison D ◻ Inclinaison G →<br>**Lèvres au repos :**<br>◻ Contact normal ◻ Lèvres sans contact<br>◻ Contact hypertonique (serrage)<br>→ **Tonus des masséters au repos :** ◻ Relâché (normal) ◻ Hypertonie<br>◻ Hypotonie ◻ Contracté-relâché<br>→ **Parésie éventuelle :** ◻ faciale ◻ labiale ◻ pas de parésie<br>+ *côté* : ◻ gauche ◻ droite →<br>**Ouverture des yeux :**<br>◻ Deux yeux<br>◻ Ouverture possible des deux yeux mais un œil ouvert préférentiellement : ◻ G ◻ D<br>◻ Ouverture d'un seul œil possible<br>→ **Mouvements spontanés de la tête** :<br>◻ oui ◻ non	

		→ **Respiration au repos** : ◻ buccale ◻ nasale ◻ trachéotomie	

**3. Stimulations externes de la sphère orofaciale**	→ **Mobilisation passive de la tête** : poser une main ouverte au niveau de la base du crâne et tenter de mobiliser légèrement la tête dans les différentes directions<br>→ **Soutien de la tête** : retirer le coussin ou relever la tête si elle est en flexion et noter si le soutien de la tête est possible pendant les 10 secondes qui suivent	→ **Mobilité passive de la tête** :<br>◻ Possible sans résistance<br>◻ Possible mais avec résistance et tensions ressenties<br>◻ Impossible<br>→ **Soutien de la tête** :<br>◻ Satisfaisant<br>◻ Possible mais bref (<3sec)<br>◻ Impossible	

	→ **Pressions fermes** *(voir annexe n°4)* : réaliser une série de pressions fermes - relâchements de pression à un bon rythme, paumes ouvertes au niveau de la face = front-sommet du crâne, deux tempes, œil-sommet du crâne, deux joues, bouche-sommet du crâne<br>Des exemples de manifestations d'incommodités pourraient être des grimaces, des tentatives d'évitement du contact, etc.<br><br>→ **Toucher léger** *(voir annexe n°4)* : effleurer avec l'index différentes zones de la face, en espaçant chaque stimulation de 5 secondes = tempes vers front, joues, menton, lèvre supérieure, lèvre inférieure.<br>Des exemples de manifestations d'incommodités pourraient être le fait de coller sa langue au palais.<br><br>NB : A prendre en compte dans l'évaluation du profil de sensibilité	→ **Pressions fermes :**<br>☐ Aucune réaction<br>☐ Manifestation d'incommodité<br>+ Préciser le type de manifestation :<br>……………………………………………………………………<br><br>→ **Toucher léger** :<br>☐ Aucune réaction<br>☐ Manifestation d'incommodité<br>+ Préciser le type de manifestation :<br>……………………………………………………………………	
**4. Ouverture buccale**	🕓 **Item O1. Initiation d'ouverture buccale :**<br>1. Demander au patient « Ouvrez la bouche » 3 fois, en laissant minimum 10 secondes entre chaque demande. Le niveau 3 est atteint si le patient ouvre la bouche lors de minimum 2 demandes.<br>2. En l'absence de réaction à la commande d'ouverture buccale, approcher une cuillère de sa bouche – à répéter 3 fois si le patient ne réagit pas au bout de 10 secondes<br>3. En l'absence de réaction 10 secondes après la 3ème approche de la cuillère, faire des allers-retours avec la cuillère puis avec l'index sur sa lèvre inférieure<br>4. En l'absence de réaction au bout de 10 sec, appuyer avec le pouce sur sa mâchoire inférieure (aide active)<br><br>Si le patient a une ouverture buccale constante, on recherchera une réaction d'ouverture buccale plus grande à l'approche de la cuillère. En l'absence de cette réaction, on cotera le niveau 0.<br><br>**NB** : Si le patient n'ouvre pas sa bouche sur aide active du thérapeute, on tiendra compte de cette information pour l'évaluation du profil de tonicité en « T+ hypercontraction »	☐ *Niveau 3* : Ouverture buccale sur commande (min 2/3)<br>☐ *Niveau 2* : Ouverture buccale à l'approche de la cuillère<br>☐ *Niveau 1* : Ouverture buccale sur stimulation labiale<br>☐ *Niveau 0* : Ouverture de la bouche impossible ou seulement sur aide active du thérapeute	Cuillère vide<br>Chronomètre

	**Amplitude d'ouverture buccale** : pour estimer si le passage d'aliments est possible, vérifier que cette ouverture buccale (obtenue à l'item O1) est au moins égale à 2 doigts (index et majeur)	**Amplitude d'ouverture buccale** :<br>☐ égale à 2 doigts<br>☐ supérieure à 2 doigts<br>☐ inférieure à 2 doigts	

5. **Observations intra-buccales**	Décrire **l'hygiène buccale et l'état dentaire** du patient	→ **Hygiène buccale** :<br>◻ satisfaisante ◻ sécheresse ◻ chargée<br>→ **Décrire la dentition :** ◻ Dentition complète<br>◻ Edentition totale ◻ Edentition partielle + préciser :<br>→ **Etat dentaire** :<br>◻ Correct ◻ Tartre ◻ Gingivite ◻ Saignements<br>◻ Dents « limées » ◻ Bruxisme ◻ Résidus alimentaires<br>→ **Prothèse dentaire** : ◻ oui ◻ non<br>→ **Prothèse pour bruxisme** : ◻ oui ◻ non<br>→ **Présence de bavage** : ◻ oui ◻ non<br>**Si oui** : ◻ gauche ◻ droite	Lampe de poche
	**Item O2. Sécrétions endo-buccales :**<br>Observer la présence et la quantité de sécrétions salivaires ou bronchiques sèches ou humides dans la bouche du patient : dans les joues, les sillons labiaux, sur le plancher de bouche, sur la langue et dans l'oropharynx. On détermine si la quantité de sécrétions salivaires et bronchiques en bouche par rapport à la taille de la cavité orale est plutôt de l'ordre de 0-20%, de 2080% ou de 80-100% ou si elles sont absentes.<br><br>NB : si aucune ouverture buccale n'est possible, tenter d'observer la présence éventuelle de sécrétions salivaires à d'autres moments (lors de bâillements par exemple). Noter 0 si l'item n'a pas pu être évalué.	◻ *Niveau 3* : Bouche humide mais sans sécrétions significatives<br>◻ *Niveau 2* : Peu de sécrétions visibles (0-20%)<br>◻ *Niveau 1* : Sécrétions en quantité modérée (20-80%)<br>◻ *Niveau 0* : Sécrétions en quantité significative (80-100%)	Lampe de poche
	Décrire **l'aspect** des sécrétions salivaires ou bronchiques intra-buccales : texture, couleur, etc.	**Aspect des sécrétions** :<br>…………………………………………………..	
**6. Initiation du réflexe de déglutition salivaire**	🕓 **Item P1. Initiation du réflexe de déglutition salivaire :** 1. Demander au patient « Avalez votre salive », en faisant un mouvement exagéré de tête et en plaçant notre main sur notre gorge pour indiquer le mouvement du larynx. Faire cette demande 3 fois, en laissant minimum 10 secondes entre chaque demande.<br>Le niveau 3 est atteint si le patient déglutit lors de minimum 2 demandes.<br>2. En l'absence de réaction à la commande, passer à l'item « P2. Latence de déclenchement du réflexe de déglutition sur stimulation ». Puis coter l'item P1 sur la base des déglutitions sur stimulation observées à l'item P2, ainsi que sur la base des déglutitions salivaires observées pendant l'examen.	◻ *Niveau 3* : Déglutition de salive sur commande (min 2/3)<br>◻ *Niveau 2* : Déglutition de salive spontanée et sur stimulation<br>◻ *Niveau 1* : Déglutition de salive uniquement sur stimulation<br>◻ *Niveau 0* : Pas de déglutition de salive que ce soit sur stimulation ou spontanément	Chronomètre
	Le cas échéant, préciser si la commande verbale a **amorcé une déglutition non aboutie** (mouvements de langue ou ascensions laryngées sans déclenchement de la déglutition) :	◻ Déglutition amorcée par la commande verbale mais non aboutie	

**7. Déclenchement du réflexe de déglutition salivaire sur stimulation**	🕓 **Item P2. Latence de déclenchement du réflexe de déglutition salivaire sur stimulation :**<br>Faire trois allers-retours de gauche à droite avec un coton-tige imbibé d'une solution sucrée et froide sur les différentes zones, dans l'ordre suivant : 1) Apex lingual, 2) Base de langue, 3) Voile du palais, 4) Mur postérieur pharyngé<br>**NB :** si aucune ouverture buccale n'est possible, stimuler les lèvres.<br>Chronométrer le temps de réaction après chaque stimulation. Si aucun mouvement labial ou lingual ni déglutition ne survient après une stimulation, **attendre 10 secondes** pour passer à la zone suivante. Si un mouvement labial ou lingual survient suite à la stimulation, **attendre 30 secondes** maximum puis passer à la zone suivante.<br><br>NB : On arrête le chronomètre quand le patient déclenche une déglutition aboutie (et pas uniquement des mouvements).<br>Noter le **temps** mis pour le déclenchement de la déglutition dans cette zone en P2. Noter quelles zones ont déclenché une déglutition et quelles zones ont amorcé une déglutition non aboutie (élévation laryngée en plusieurs à coups, début de propulsion linguale, etc).<br><br>**Critère d'arrêt** de cotation de l'item **:**<br>1. Déclenchement d'un réflexe de déglutition<br>2. Survenue d'un réflexe nauséeux<br>3. Pas de réaction après stimulation au niveau du mur postérieur pharyngé	☐ *Niveau 3* : entre 0 et 5 secondes<br>☐ *Niveau 2* : entre 5 et 10 secondes<br>☐ *Niveau 1* : > 10 sec<br>☐ *Niveau 0* : Pas de déclenchement ou non-réalisable<br><br>**Zone(s) ayant déclenché une déglutition :**<br>☐ Lèvres ☐ Apex lingual<br>☐ Base de langue ☐ Voile du palais<br>☐ Mur postérieur pharyngé<br>☐ Absence de déglutition aboutie<br><br>**Zone(s) ayant amorcé une déglutition non aboutie** :<br>☐ Lèvres ☐ Apex lingual<br>☐ Base de langue ☐ Voile du palais<br>☐ Mur postérieur pharyngé<br>☐ Absence de déglutition non aboutie	Coton-tige imbibé d'une solution sucrée et froide<br><br>Chronomètre
	Si une déglutition se déclenche au cours des stimulations de l'item P2 mais pas un réflexe nauséeux, continuer les stimulations de l'item P2 pour rechercher la présence d'un **réflexe nauséeux et sa zone de déclenchement**.<br><br>NB : A prendre en compte dans l'évaluation du profil de sensibilité	**Réflexe nauséeux** : ☐ oui ☐ non<br>*Si oui, zone ayant déclenché le réflexe nauséeux :*<br>☐ Lèvres ☐ Apex lingual<br>☐ Base de langue ☐ Voile du palais<br>☐ Mur postérieur pharyngé<br><br>*Autres signes d'incommodité éventuels :*<br>……………………………………………………………………	Coton-tige imbibé d'une solution sucrée et froide
	En cas de déglutition, noter la **qualité de l'ascension laryngée**	**Ascension laryngée :** ☐ Présente ☐ Absente<br>☐ Présente anormalement : O incomplète O ralentie O en plusieurs à-coups	

**8. Test fonctionnel**	🕓 **Items O3. Préhension labiale et O4. Propulsion linguale :**<br>• Préparer une cuillère à café contenant 5mL de solution colorée modérément épaissie (texture IDDSI3) froide (NB : 5 mL = 1 cuillère à café).<br>• Reproposer le protocole d'initiation de l'ouverture buccale (item O1) et présenter la cuillère préparée.<br>• Observer la préhension labiale de la cuillère (**item O3**). Si le patient ne réagit pas, le stimuler verbalement en disant : « Prenez la cuillère ».<br>• S'il n'y a pas d'ouverture buccale et de préhension labiale à l'approche de la cuillère mais qu'il est possible d'entrouvrir la bouche passivement, déposer le contenu de la cuillère sur la langue et observer les réactions.<br>• Lancer le chronomètre au moment de la préhension labiale ou quand le liquide est déposé sur la langue.<br>• Observer la propulsion linguale (**item O4**), en plaçant une main sous le plancher buccal du patient pour sentir les éventuels mouvements de la langue.<br>• Vérifier la présence éventuelle de stases buccales post-déglutition.<br><br>Si le patient ne présente aucune réaction labiale ou linguale **au bout de 10 secondes**, coter 0.<br>Si le patient présente une préhension labiale complète (niveau 3 de l'item O3), refaire deux essais avec une cuillère vide pour vérifier la stabilité de la préhension. Si le patient réussit toujours cet item, coter niveau 3 ; si le patient échoue au 2e ou au 3e essai, coter niveau 2.<br><br>**ATTENTION : si le patient ne présente aucune déglutition salivaire sur stimulation (niveau 0 à l'item P1), qu'aucune déglutition spontanée n'est observée ou qu'il a un réflexe de morsure important ou un trismus, le test fonctionnel ne sera pas réalisé.** Dans ce cas, l'item « propulsion linguale » sera coté 0 et l'item « préhension labiale » sera testé avec une cuillère vide.<br><br>**NB :** De même, si le test fonctionnel ne peut être réalisé car aucune ouverture buccale n'est possible, les items « propulsions linguale » et « préhension labiale » seront cotés 0.	**O3. Préhension labiale :**<br>☐ *Niveau 3* : Préhension labiale correcte et spontanée systématiquement<br>☐ *Niveau 2* : Préhension labiale adéquate mais nonsystématique ou uniquement sur stimulation verbale<br>☐ Niveau 1 : Préhension labiale incomplète en spontané ou sur stimulation verbale<br>☐ *Niveau 0* : Aucune préhension labiale (pas de réaction ou serrage des lèvres)<br><br>**O4. Propulsion linguale :**<br>☐ *Niveau 3* : Propulsion linguale adéquate<br>☐ *Niveau 2* : Propulsion linguale pathologique avec présence possible de stases post-déglutition<br>☐ *Niveau 1* : Quelques mouvements linguaux mais insuffisants pour propulser le bolus<br>☐ *Niveau 0 :* Aucun mouvement lingual : passage passif du bolus au niveau pharyngé, stagne en bouche ou ressort en bavage	Chronomètre<br><br>Cuillère à café 5mL de solution colorée épaissie froide texture IDDSI3 (modérément épais)

	En plus des items de préhension et de propulsion linguale ci-dessus, noter la présence éventuelle d'un **déclenchement d'une déglutition et/ou d'une toux et/ou d'une fausse route.**	→ **Déclenchement d'un réflexe de déglutition** :<br>□ oui □ non<br>→ **Temps de déclenchement après que le liquide ait touché la langue** : … sec<br>→ **Déglutitions multiples :** □ oui □ non<br>→ **Episode de toux** : □ oui □ non<br>*Si oui* : □ avant déglutition □ après déglutition<br>□ à distance<br>→ **Signes d'encombrement majoré ou dyspnée** :<br>□ oui □ non<br>→ **Autres signes de fausse route :**<br>…………….........................	
	Noter les caractéristiques du déclenchement de **mouvements linguaux** :	**Mouvements linguaux spontanés :**<br>→ *En dehors des tentatives de déglutition* : □ oui □ non<br>→ *Lors des tentatives de déglutitions salivaires :* □ oui □ non → *En réponse à stimulation du visage, des lèvres ou de la langue elle-même* : □ oui □ non<br>+ *Si oui*, préciser la stimulation qui a induit les mouvements linguaux :<br>………………………………..	
	Noter la présence d'éventuels **réflexes archaïques** à l'ouverture de la bouche et à la préhension labiale :	→ **Réflexe de morsure :** □ oui □ non<br>→ **Réflexe de succion :** □ oui □ non<br>→ **Réflexe de mâchonnement :** □ oui □ non	
	En cas de déglutition, noter la **qualité de l'ascension laryngée**	**Ascension laryngée :**<br>□ Présente □ Absente<br>□ Présente anormalement : O incomplète O ralentie O en plusieurs à-coups	
	**Item P3. Trachéotomie :**<br>Questionner l'équipe soignante, regarder dans le dossier du patient et/ou observer la trachéotomie du patient.	□ *Niveau 3* : Trachéotomie en cours de sevrage, possibilité de boucher la canule au doigt ou absence de trachéotomie □ *Niveau 2* : Trachéotomie sans ballonnet ou avec ballonnet dégonflé en permanence<br>□ *Niveau 1* : Trachéotomie avec ballonnet en cours de sevrage<br>□ *Niveau 0* : Trachéotomie avec ballonnet gonflé<br>*Si pas de trachéotomie*, le patient en a-t-il eu une dans le passé : □ oui □ non	Dossier du patient

**9. Respiration**	**Item P4. Respiration et encombrement haut et bas** :<br>Questionner l'équipe soignante, regarder dans le dossier du patient et/ou observer le patient.<br><br>Pour évaluer le niveau d'encombrement haut et bas, repérer la **sévérité des signes d'encombrement** suivants chez le patient : respiration bruyante ou sifflante, toux laissant percevoir la présence de mucus, etc. Il convient également de prendre en compte la **quantité d'aspirations intrabuccales et/ou endotrachéales** réalisées par l'équipe soignante ou le patient lui-même. Enfin, le **besoin d'un suivi en kinésithérapie respiratoire** est à prendre en compte.	□ *Niveau 3* : pas d'encombrement<br>□ *Niveau 2* : peu d'encombrement<br>□ *Niveau 1* : encombrement modéré<br>□ *Niveau 0* : bronchopneumonies fréquentes ou encombrement important	Dossier du patient
	En cas **d'encombrement**, préciser son type.	→ **Systèmes aidant la respiration** : □ humidificateurs □ oxygénation Si oxygénation, nombre de litres ou % :<br>………………………………<br>→ **Encombrement haut** :□ oui □ non – préciser :<br>……………………………………………………………………………<br>→ **Encombrement bas** :□ oui □ non – préciser :<br>……………………………………………………………………………<br>→ **Fréquence des aspirations :**<br>……………………………………………………………………………<br>→ **Fréquence de la kiné respiratoire :**<br>……………………………………………………………………………	
**10. Voix Articulation Langage**	Noter la capacité à produire des **bruits spontanés, des mots articulés ou une communication fiable** (d'après la SECONDs, réponse correcte à 5 questions autobiographiques simples et fermées) :	→ **Bruits/sons spontanés** : □ oui □ non<br>→ **Mots articulés** : □ oui □ non<br>→ **Communication fiable** :□ oui □ non	
**11. Profils de tonicité et de sensibilité**	**Profil de tonicité** :<br>Observer l'aspect facial du patient au repos, lors des stimulations tactiles et lors des essais d'initiation d'ouverture buccale	□ *Profil T-* : *hypotonicité* : ouverture buccale en continu, bavage<br>□ *Profil T+* : *hypercontraction/serrage* : serre les dents en continu et/ou lors des stimulations tactiles, résiste à l'ouverture buccale<br>□ *Profil T neutre* : absence de signes d'hypotonicité ou d'hypertonicité	

	**Profil de sensibilité** :<br>Observation des réactions aux stimulations externes et internes de la sphère oro-faciale, et du réflexe nauséeux	◻ *Profil S-* : *hyposensibilité* : absence de réaction aux stimulations tactiles, réflexe nauséeux faible ou absent<br>◻ *Profil S+* : *hypersensibilité* : sursauts, grimaces lors des stimulations tactiles de toucher léger, réflexe nauséeux exacerbé<br>◻ *Profil S neutre* : absence de signes d'hyposensibilité ou d'hypersensibilité	

**SWADOC-scored total : /24**	**SWADOC-scored phase orale : /12**<br>**SWADOC-scored phase pharyngée : /12**	**Profil de tonicité : T+ / Tn / T-**<br>**Profil de sensibilité : S+ / Sn / S-**
**Remarques :**		

ANNEXES

Annexe 1 : items traduits de la *Food Intake Level Scale* (FILS)[2]

Pas d'alimentation orale
Niveau 1 : pas de stimulation de la déglutition en dehors des soins buccaux.
Niveau 2 : stimulations de la déglutition « à vide » (sans nourriture)
Niveau 3 : stimulations de la déglutition utilisant une petite quantité de nourriture
Combinaison d'alimentation orale et alimentation entérale
Niveau 4 : alimentation en texture facile et en quantité moindre qu'un repas (alimentation plaisir) ingérée oralement.
Niveau 5 : alimentation en texture facile ingérée oralement correspondant à un ou deux repas mais une alimentation complémentaire non-orale est aussi donnée.
Niveau 6 : alimentation en texture facile reçue à trois repas mais une alimentation non-orale est aussi donnée en complément.
Alimentation orale uniquement
Niveau 7 : alimentation en texture facile aux trois repas. Pas d'alimentation complémentaire non-orale.
Niveau 8 : le patient ingère trois repas en excluant les aliments qui sont particulièrement difficile à avaler.
Niveau 9 : le patient ingère trois repas sans restriction au niveau des textures mais des conseils médicaux sont donnés. Niveau 10 : alimentation orale ordinaire aux trois repas.

Remarques sur les termes employés :
- Stimulations de la déglutition : stimulations réalisées par un thérapeute compètent dans le domaine, un soignant formé ou le patient luimême pour améliorer sa déglutition.
- Alimentation en texture facile : aliments qui sont préparés de telle manière qu'ils sont faciles à déglutir même sans mastication par exemple de la viande et des légumes sous forme pâteuse ou homogénéisée dans un mixer.
- Alimentation non-orale : gastrostomie, sonde naso-gastrique ou alimentation parentérale.
- Aliments qui sont particulièrement difficiles à avaler : aliments secs et fragmentables, aliments durs, eau, etc.
- Conseils médicaux : conseils donnés dans le but de limiter les symptômes dysphagiques comme les épisodes de toux ou la sensation d'aliments stagnant dans le pharynx.

Annexe 2 : standardisation internationale des textures adaptées à la dysphagie (IDDSI)[1]

Pour les scores à la FILS correspondant, nous noterons les textures d'aliments et d'hydratation proposées aux patients en suivant la terminologie internationale IDDSI (International Dysphagia Diet Standardisation Initiative).

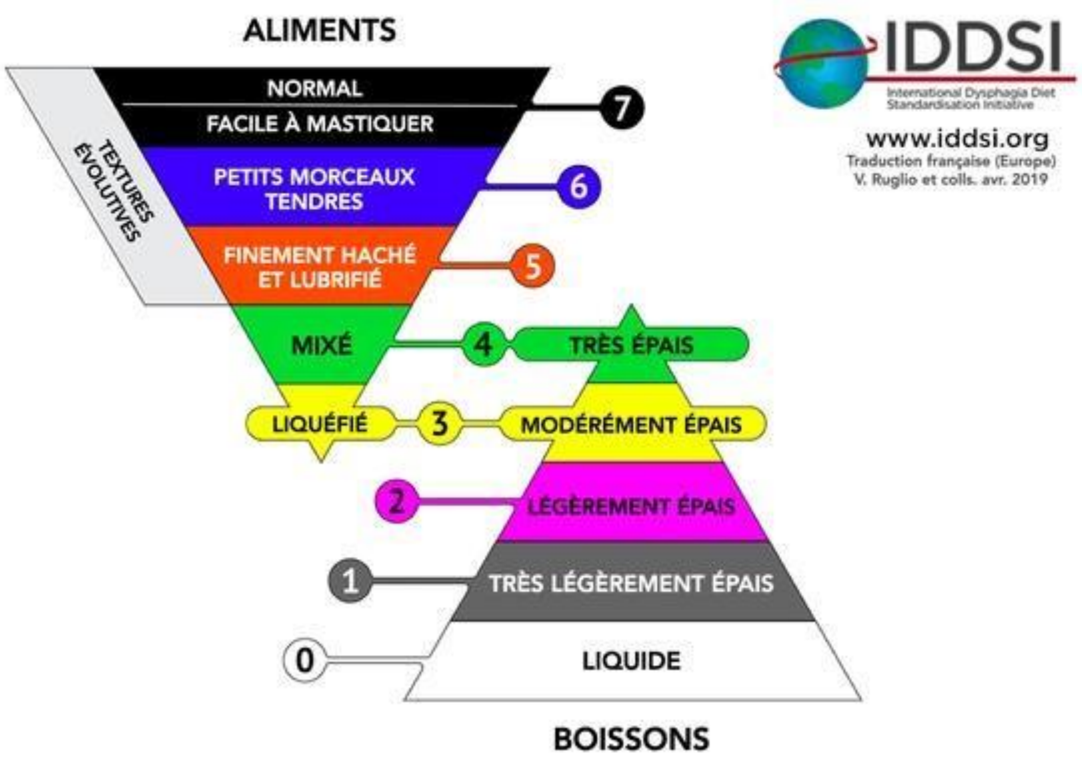

Annexe 3 : protocole d'éveil de la *Coma Recovery Scale Revised* (CRS-R)[5,6]

PROTOCOLE DE STIMULATION D'EVEIL ©2008
**INDICATIONS**
1) Le but de cette intervention est de prolonger la durée d'éveil du patient (ouverture des yeux).<br>2) Ce protocole doit être administré à chaque fois que le patient :<br>- Démontre des périodes prolongées de fermeture des yeux ET/OU<br>- Arrête de répondre aux questions pendant 1 minute ou plus<br>3) Recommencer le protocole de simulation d'éveil quand :<br>- Une fermeture prolongée des yeux se reproduit ET/OU<br>- Les réponses comportementales arrêtent malgré une ouverture spontanée des yeux
**INTERVENTIONS**
**Pression profonde** :<br>1) Effectuez une pression profonde unilatérale au niveau du visage, la nuque, la main, le torse, le dos, la jambe, et les orteils. Pendant que vous pincez le muscle de manière intense vous le « roulez » 3 à 4 fois entre l'index et le pouce. Cette procédure doit être répétée de manière séquentielle dans le sens du visage vers les orteils. Assurez-vous qu'il n'y ait pas de lignes internes, lésions locales (fractures, contusions, ulcérations) avant d'entamer la pression profonde.<br>2) Répéter la procédure du côté controlatéral.

Annexe 4 : stimulations externes de la sphère oro-faciale

Objectif :
Ce sous-test évalue les réactions du patient à des stimulations tactiles faciales. Il vise notamment à déterminer le « profil » de sensibilité et de tonus du patient, en réaction aux stimulations afin d'orienter la prise en charge du patient.

Ce subtest s'est inspiré du sous-test de la sensibilité oro-faciale de la Comprehensive Assessment Measure for the Minimally Responsive Individual (CAMMRI)[7] pour les stimulations légères et des méthodes de stimulation des dysoralités sensorielles de Catherine Senez[8] pour les stimulations fermes (tour de la maison).

Matériel :
- Gants en latex/vinyl

Administration du test :
Dites au patient que vous allez toucher des zones de son visage.
Suivez l'ordre spécifique du formulaire de données concernant les zones à cibler. Faites d'abord le test de la pression ferme puis celui du toucher léger.

1) Pressions fermes

En utilisant les deux mains, paumes ouvertes et posées sur la tête du patient, il faut réaliser une série de contacts, pressions, lâchés de pression, retraits. Les pressions devront se succéder suivant un bon rythme et très rapidement. La pression appliquée doit être modérée à ferme en un endroit de la zone à stimuler (pas d'effleurement de toute la zone). Description de la stimulation :

- Front-sommet du crâne
- Les deux tempes
- Un œil-sommet du crâne (creuser la main pour ne pas toucher l'œil)
- L'autre œil-sommet du crâne
- Les deux joues
- La bouche-sommet du crâne

2) Toucher léger

Pendant les stimulations légères, effectuez des effleurements avec l'index. Ne touchez qu'une seule fois chaque zone d'application. Espacez chaque stimulation de 5 secondes.

**Zones à stimuler**	**Description de la stimulation**
a) Tempes/front	Utiliser une caresse continue de la tempe jusqu'au centre du visage, un côté puis l'autre
b) Joues	Commencer au niveau du milieu de l'oreille, suivre l'os de la joue et s'arrêter dans la zone du milieu de l'œil
c) Menton	Index à plat, remonter le long du menton
d) Lèvre supérieure	Caresser la lèvre de gauche à droite
e) Lèvre inférieure	Caresser la lèvre de gauche à droite

Références

1. Cichero JAY, Lam P, Steele CM, et al. Development of International Terminology and Definitions for Texture-Modified Foods and Thickened Fluids Used in Dysphagia Management: The IDDSI Framework. *Dysphagia*. 2017;32(2):293-314. doi:10.1007/s00455-016-9758-y

2. Kunieda K, Ohno T, Fujishima I, Hojo K, Morita T. Reliability and Validity of a Tool to Measure the Severity of Dysphagia: The Food Intake LEVEL Scale. *Journal of Pain and Symptom Management*. 2013;46(2):201-206. doi:10.1016/j.jpainsymman.2012.07.020

3. Aubinet C, Cassol H, Bodart O, et al. Simplified Evaluation of CONsciousness Disorders (SECONDs) : A new tool to assess consciousness in severely brain-injured patients. *Annals of physical and rehabilitation medicine*. Published online 2020. doi:10.1016/j.rehab.2020.09.001

4. Sanz LRD, Aubinet C, Cassol H, et al. SECONDs administration guidelines: A fast tool for assessing consciousness in brain-injured patients Leandro R. D. Sanz[1],[2]*, Charlène Aubinet[1],[2]*, Helena Cassol[1],[2], Olivier Bodart[1],[2], Sarah Wannez[1],[2], Estelle A. C. Bonin[1],[2], Alice Barra[1],[2], Nicolas Lejeune1,2,3,4, Charlotte Martial[1],[2], Camille Chatelle[1],[2], Didier Ledoux5,6, Steven Laureys[1],[2], Aurore Thibaut[1],[2]#, Olivia Gosseries[1],[2]#. *Journal of Visualized Experiments*. 2020;In press.

5. Giacino JT, Kalmar K, Whyte J. The JFK Coma Recovery Scale-Revised: Measurement characteristics and diagnostic utility. *Archives of Physical Medicine and Rehabilitation*. 2004;85(12):2020-2029. doi:10.1016/j.apmr.2004.02.033

6. Schnakers C, Majerus S, Giacino J, et al. A French validation study of the Coma Recovery Scale-Revised (CRS-R). *Brain Injury*. 2008;22(10):786-792. doi:10.1080/02699050802403557

7. Gollega A, Meghji C, Renton S, et al. Multidisciplinary assessment measure for individuals with disorders of consciousness. *Brain Injury*. 2015;29(12):1460-1466. doi:10.3109/02699052.2015.1071426

8. Senez C. *Rééducation des troubles de l’oralité et de la déglutition*. 2nd ed. De Boeck Supérieur; 2015.
