# Supplementary material for: Swallowing Assessment in Post-Comatose Patients: A Feasibility Study on the SWADOC Tool
Source: J Clin Med. 2024 May 31;13(11):3268. doi: 10.3390/jcm13113268 (PMC11173236; doi:10.3390/jcm13113268)
Supplement: Supplementary file 1 [file jcm-13-03268-s001.zip › jcm-2964910-TableS1.pdf]

Table S1 - Individual demographic and clinical characteristics including SECONDs and detailed SWADOC scores.

**Diagnosis**	**UWS**								**MCS-**														**MCS+**										**EMCS**					
**Patients**	P7		P9			P13			P1			P10			P11			P12			P14		P2			P4		P5			P8			P3			P6		
Gender	F		F			M			F			F			M			M			M		M			M		F			M			M			M		
Age (years)	43		55			55			51			65			66			58			67		48			59		64			61			42			49		
TSI (months)	2.6		134.2			87.9			8.7			54.4			103.4			108.4			117.7		10.9			8.9		8.3			2.9			10.7			13.7		
Etiology	V		A			A			V			T			T			T			V		A			T		V			T			E			T		
Trachostomy with cuff	Y		N			N			Y			N			N			N			N		N			Y		N			N			N			N		
FILS score	1		1			1			2			1			1			1			1		3			2		2			2			3			9		
**Visits**	1	2	1	2	3	1	2	3	1	2	3	1	2	3	1	2	3	1	2	3	1	2	1	2	3	1	2	1	2	3	1	2	3	1	2	3	1	2	3
**SECONDs score**	1	1	1	1	1	1	1	1	4	4	4	4	4	4	4	4	4	1	4	3	5	5	7	6	6	6	7	6	6	6	6	3	4	8	8	8	8	8	8
**SWADOC:**																																							
Item O1	**0**	0	0	**0**	0	0	**0**	0	**0**	0	0	0	0	**0**	2	2	**2**	**0**	0	0	**0**	0	**3**	3	3	**3**	3	0	**0**	0	0	0	**0**	3	**3**	3	2	2	2
Item O2	**1**	1	2	**1**	1	2	**2**	2	**3**	3	3	2	2	**1**	1	3	**3**	**3**	1	1	**2**	3	**3**	3	3	**3**	3	3	**3**	2	3	3	**3**	0	**1**	1	3	3	3
Item O3	**0**	0	0	**0**	0	0	**0**	0	**0**	0	0	0	0	**0**	0	0	**0**	**0**	0	0	**3**	0	**2**	1	0	**1**	1	0	**0**	0	0	0	**0**	3	**3**	3	3	3	3
Item O4	**0**	0	0	**0**	0	0	**0**	0	**1**	1	1	1	0	**0**	0	0	**0**	**0**	0	0	**2**	0	**2**	1	1	**1**	0	1	**1**	0	1	0	**1**	3	**3**	2	3	3	3
**Oral subscore**	**1**	1	2	**1**	1	2	**2**	2	**4**	4	4	3	2	**1**	3	5	**5**	**3**	1	1	**7**	3	**10**	8	7	**8**	7	4	**4**	2	4	3	**4**	9	**10**	9	11	11	11
Item P1	**2**	2	1	**1**	1	0	**2**	0	**2**	2	2	1	1	**2**	0	0	**0**	**0**	0	0	**2**	2	**3**	3	3	**3**	3	3	**3**	3	2	2	**2**	2	**3**	3	2	2	2
Item P2	**3**	2	0	**0**	0	0	**2**	0	**2**	1	1	1	2	**1**	0	0	**0**	**0**	0	0	**2**	2	**0**	2	1	**2**	0	2	**3**	1	2	3	**3**	1	**3**	1	1	1	1
Item P3	**1**	1	2	**2**	2	2	**2**	2	**0**	0	0	3	3	**3**	3	3	**3**	**3**	3	3	**2**	2	**3**	3	3	**0**	0	3	**3**	3	3	3	**3**	3	**3**	3	3	3	3
Item P4	**2**	2	1	**2**	0	3	**2**	1	**3**	3	3	2	1	**3**	1	1	**1**	**3**	3	3	**3**	1	**3**	2	3	**0**	0	1	**1**	2	3	3	**3**	1	**2**	1	3	3	3
**Pharyngeal subscore**	**8**	7	4	**5**	3	5	**8**	3	**7**	6	6	7	7	**9**	4	4	**4**	**6**	6	6	**9**	7	**9**	10	10	**5**	3	9	**10**	9	10	11	**11**	7	**11**	8	9	9	9
**Total score**	**9**	8	6	**6**	4	7	**10**	5	**11**	10	10	10	9	**10**	7	9	**9**	**9**	7	7	**16**	10	**19**	18	17	**13**	10	13	**14**	11	14	14	**15**	16	**21**	17	20	20	20

A: anoxic; E: epileptic; EMCS: emergence of minimal conscious state; F: female; FILS: Food Intake Level Scale; M: male; MCS-: minimal conscious state minus; MCS+: minimal conscious state plus; N: no; SECONDs: Simplified Evaluation of CONsciousness Disorders; SWADOC: Swallowing Assessment in Disorders Of Consciousness; T: traumatic; Trach.: tracheostomy; TSI: time since insult; UWS: unresponsive wakefulness syndrome; V: vascular; Y: yes.In bold: scores from the best total score assessment, used to measure the test-retest reliability.
